# A role for Insulin-like growth factor 2 in specification of the fast skeletal muscle fibre

**DOI:** 10.1186/1471-213X-7-65

**Published:** 2007-06-08

**Authors:** Deborah Merrick, Tao Ting, Lukas Kurt Josef Stadler, Janet Smith

**Affiliations:** 1 School of Biosciences, University of Birmingham, Edgbaston, Birmingham, B15 2TT, UK; 2 School of Biomedical Sciences, Nottingham University Medical School, Queen's Medical Centre, Nottingham NG7 2UH, UK; 3 Geriatric department, Ruijin Hospital, Shanghai Second Medical University, 200025, Shanghai, People's Republic of China

## Abstract

**Background:**

Fibre type specification is a poorly understood process beginning in embryogenesis in which skeletal muscle myotubes switch myosin-type to establish fast, slow and mixed fibre muscle groups with distinct function. Growth factors are required to establish slow fibres; it is unknown how fast twitch fibres are specified. Igf-2 is an embryonically expressed growth factor with established *in vitro *roles in skeletal muscle. Its localisation and role in embryonic muscle differentiation had not been established.

**Results:**

Between E11.5 and E15.5 fast Myosin (FMyHC) localises to secondary myotubes evenly distributed throughout the embryonic musculature and gradually increasing in number so that by E15.5 around half contain FMyHC. The Igf-2 pattern closely correlates with FMyHC from E13.5 and peaks at E15.5 when over 90% of FMyHC+ myotubes also contain Igf-2. Igf-2 lags FMyHC and it is absent from muscle myotubes until E13.5. Igf-2 strongly down-regulates by E17.5. A striking feature of the FMyHC pattern is its increased heterogeneity and attenuation in many fibres from E15.5 to day one after birth (P1). Transgenic mice (MIG) which express Igf-2 in all of their myotubes, have increased FMyHC staining, a higher proportion of FMyHC+ myotubes and loose their FMyHC staining heterogeneity. In Igf-2 deficient mice (MatDi) FMyHC+ myotubes are reduced to 60% of WT by E15.5. *In vitro*, MIG induces a 50% excess of FMyHC+ and a 30% reduction of SMHyC+ myotubes in C2 cells which can be reversed by Igf-2-targeted ShRNA resulting in 50% reduction of FMyHC. Total number of myotubes was not affected.

**Conclusion:**

In WT embryos the appearance of Igf-2 in embryonic myotubes lags FMyHC, but by E15.5 around 45% of secondary myotubes contain both proteins. Forced expression of Igf-2 into all myotubes causes an excess, and absence of Igf-2 suppresses, the FMyHC+ myotube component in both embryonic muscle and differentiated myoblasts. Igf-2 is thus required, not for initiating secondary myotube differentiation, but for establishing the correct proportion of FMyHC+ myotubes during fibre type specification (E15.5 - P1). Since specific loss of FMyHC fibres is associated with many skeletal muscle pathologies these data have important medical implications.

## Background

In mouse, skeletal muscle fibres are formed during the second half of embryogenesis (E11.5–E16.5). The dorsal epaxial muscles and the proximal and distal (hypaxial) muscles are thought to derive respectively from two distinct groups of myogenic precursors (stem cells) which originate in the somites [1]. Both groups of cells subsequently undergo two overlapping waves of differentiation which form respectively, the primary and secondary myotubes. In primary myogenesis (E9.5–E13.5) a scaffold of short, fat primary myotubes is established [2]. Secondary myogenesis, in which a larger number of long, thin secondary myotubes is formed around the primary scaffold, is initiated around E11.5 and continues into early post-natal life [3]. Primary and secondary myotubes can be distinguished by their morphology, their location and by the skeletal myosin sub-types that they produce. In cross-section, it can be seen that a number of smaller diameter secondary myotubes form in clusters around a single large diameter primary myotube [3]. Whilst both primary and secondary myotubes are reported to make development specific (foetal, embryonic and neonatal) myosins, new primary myotubes also express slow myosin whilst newly formed secondary myotubes initially express fast myosin [2,3]. Towards the end of secondary myogenesis 'fibre-type switching' takes place in which some primary myotubes become fast-myosin positive and some secondary myotubes switch to slow myosin [4,5]. This process is thought to establish discrete muscle groups with distinct function dependent upon a unique combination of fibres expressing fast (Type 2a, b and x) and slow (Type 1) myosin heavy chain (MyHC) forms and is essential for normal post-natal functioning of the skeletal musculature. Further refinement of MyHC and Myosin light chain (MyLC) expression occurs perinatally and during subsequent post-natal growth [6,7]. Mature adult mammalian skeletal muscles are uniquely defined by their particular composition of fast and slow fibre types and may express single MyHC or a mixture of several MyHC. Additional complexity is conferred by the MyLC [8].

A host of growth factors are required to establish and differentiate embryonic skeletal muscle myotubes, but very little is known about the process of fibre type switching in development. In the zebra fish embryo it has been demonstrated, that two growth factors; Hepatocyte growth factor and Myostatin play roles respectively in somitogenesis and in the establishment of the hypaxial lineage [9,10]. In mouse, several families of growth factor (Wnt, Shh and BMP) have been shown to play a role in establishing skeletal muscle lineage; whilst others (Notch, FGF family, antagonists of Wnt signalling (sFRP1, 2, 4)) play roles in maintaining, promoting or restricting skeletal muscle embryonic differentiation [11]. Commitment to the skeletal muscle lineage is reported to require the suppression of BMP4 [12]. In the chick embryo somite differentiation can be induced experimentally by several growth factors including Shh, FGF-2 and TGF-β which, in this system, act synergistically with Igf-2, Igf-1 or insulin to augment skeletal muscle differentiation although fibre type was not investigated in this study [13].

There is currently little genetic data available on the establishment of fibre type per se; however two lines of evidence suggest that this is also regulated by growth factor action. In zebra fish embryos, generation of the slow muscle fibres is dependent on Sonic hedgehog (Shh); whilst in chick limb Shh promotes slow fibre differentiation [14,15]. In mouse however Shh is associated with establishing the epaxial lineage and has not been shown to influence fibre-type selection [16]. Recently it was shown that embryonic loss of FGF-6 in the mouse results in earlier myotube formation and a skewing of fibre-type composition towards Type 1 (slow fibres) suggesting that FGF-6 may have an inhibitory effect on type 1 fibre differentiation [17]. With the exception of our data reported here, there are no other data on genetic regulation of fibre-type selection during embryonic myogenesis.

The growth factor Igf-2 can regulate differentiation in cultured skeletal muscle cells [18] and in chick somite explants [13], whilst androgenetic (paternal disomy) embryos and embryonic stem cells that over-express Igf-2 display preferential differentiation into skeletal muscle [19]. In mammals Igf-2 is reported to promote mesoderm differentiation [20]. In rodents, Igf-2 is primarily an embryonic growth factor, widely expressed throughout embryogenesis, but substantially down-regulated at birth [21]. The closely related peptide growth factor, Igf-1 has an embryonic expression pattern distinct from Igf-2 which is associated with the developing nervous system and undifferentiated mesenchyme [22-24]. Surprisingly, the protein localisation pattern for Igf-2 has not previously been established.

To establish a role for Igf-2 in fibre type specification during mammalian myogenesis we first established its precise localisation pattern in embryonic myotubes in relation to FMHyC. We showed that Igf-2 is present in 50% of embryonic myotubes at E15.5. To test the functional importance of Igf-2 localisation to particular myotubes we used a muscle targeted promoter (muscle Creatine Kinase, mCK) to force over-expression of Igf-2 in all embryonic myotubes in order to disrupt FMyHC myotube formation in these (MIG) mice. Conversely, we used Igf-2 deficient mice to establish the fate of FMyHC positive myotubes in the absence of Igf-2. By combining ShRNA interference with the MIG transgene we show that Igf-2 has a substantial impact on the generation of fast and slow MyHC containing myotubes in vitro. Fast myosin fibre-type switching is a complex and poorly understood process with important medical applications. The fast fibre-type ratio is perturbed in a number of myopathies and skeletal muscle dystrophies, whilst disproportionate fast myosin fibre degeneration is associated with skeletal muscle ageing [25,26]. Previously we have shown Igf-2 to ameliorate the dystrophic phenotype [27]. The data presented here suggest a new function for Igf-2 in the specification of this crucial muscle fibre subset.

## Results

### Immunostaining reveals a dynamic and tightly linked localisation pattern between Igf-2 and fast myosin during secondary embryogenesis

**Figure 1:**
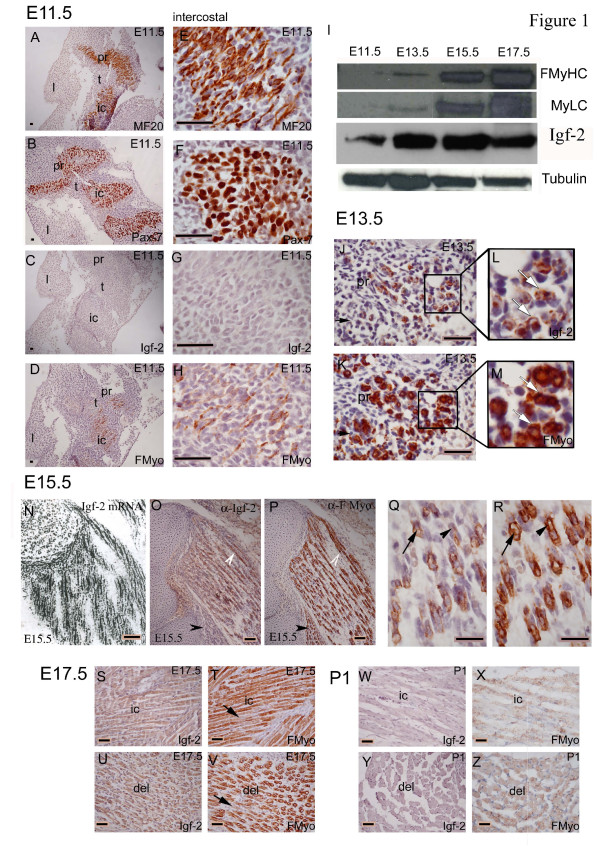
Immunostaining pattern of fast myosin and Igf-2 in the wild type (C57BL10) mouse embryo. (A, E) Pan-myosin (MF20) staining and (B, F) Pax 7 respectively, show the location of differentiated myotubes and undifferentiated myoblasts at E11.5 in intercostal (ic), limb (l), trunk muscles (t) and upper proximal limb muscles (pr); (C, G) Igf-2 is absent from E11.5 embryonic myotubes, (D, H) a small number of secondary myotubes stain with My32 (fast MyHC). (I) Immunoblot of whole embryo protein (E11.5 to E17.5) showing fast MyHC (My32), Igf-2 and tubulin. FMyHC increases with stage. Igf-2 peaks at E15.5 and is downregulated in muscle at E17.5. Igf-2 is also present in non-muscle tissues (including liver, kidney, heart, gut) between E11.5 and E17.5. (J-M, O-Z) Imunostaining pattern of Igf-2 and FMyHC in upper proximal limb muscles. (J-L) E13.5 embryos show an overlapping pattern of Igf-2 (J, L) and fast myosin (K, M) in secondary myotubes. Enlarged areas (L, M) show secondary myotubes containing both Igf-2 and fast myosin (indicated by white arrows). (N-R) At E15.5 Igf-2 and fast Myosin are localised to ~half of myotubes and show a strikingly similar pattern. (N-O) White arrows, double positive myotubes. Black arrows FMyHC positive, Igf-2 negative muscle group. (Q-R) most Igf-2 positive myotubes also stain for FMyHC (Black arrows, double positive myotubes). (N) Igf-2 mRNA is expressed predominantly in secondary myotubes broadly coincident (O) with the Igf-2 and (P) fast Myosin protein localisation patterns. (S-V) At E17.5 Igf-2 staining (S, U) is almost undetectable. Fast Myosin (T, V) staining shows increased heterogeneity compared to earlier stages (black arrows, reduced FMyHC staining). Skeletal muscles of day 1 pups (P1) contain (W, Y) no Igf-2 and (X, Z) have reduced and heterogenous fast Myosin staining (X, Z). (S-T, W-X) intercostals (ic); U-V, Y-Z upper proximal (del, deltoid) muscles. Images shown in A-D (E-H); J-K (L-M); O-P (Q-R); S, T and U, V; W, X and Y-Z are adjacent sections. Immunostaining patterns were confirmed by at least three different runs on separate embryos (many more for the Igf-2 pattern) per stage. Images shown are fully representative of each run. Comparison of FMyHC, Igf-2, Pax 7 and MF20 was achieved by staining separate adjacent sections for each antibody in the same run. Negative second antibody controls were included in each run. Peptide absorption was carried out at each stage for the Igf-2 pattern. Magnification bars indicate 10 μC on all images.

**Figure 2:**
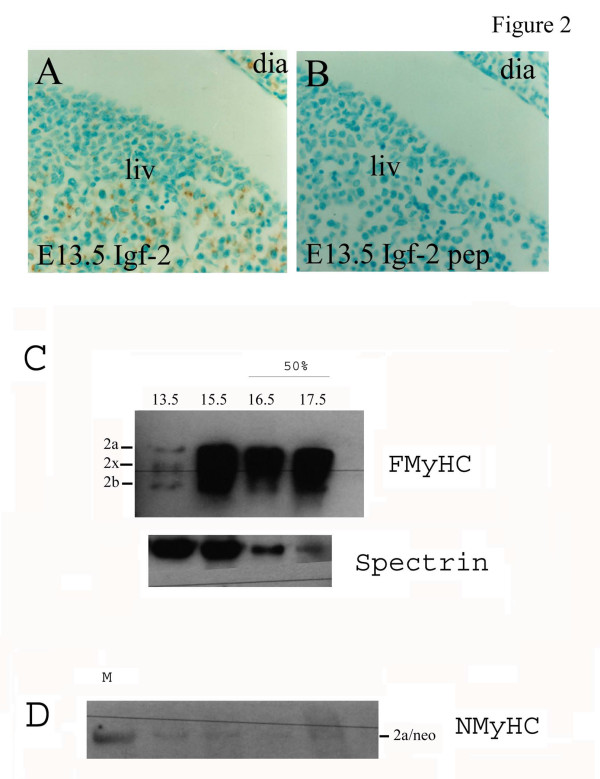
Antibody specificity; (A) Igf-2 immunostaining of adjacent sections of an e13.5 embryo illustrate positive Igf-2 staining (brown) in liver (liv) and diaphragm (dia) which, (B) is completely abolished by pre-incubation of the antibody with 2ug/ml Igf-2 (pep; peptide absorption) demonstrating the specificity of Igf-2 staining. (C-D) Immunoblotting of whole embryo protein (E12.5 – E17.5) with, (C) clone My32 anti-FMyHC antibody: detects three positive bands corresponding to the three isoforms of adult fast myosin heavy chain (FMyHC) 2b, 2a and 2x/d. E12.5 and E13.5 tracks contain 20 μg protein. E15.5 and E17.5 tracks are loaded with 10 μg protein to prevent over-exposure of FMyHC, this is reflected in the intensity of bands in the anti-Spectrin loading control. (D) Because My32 is reported to cross-react with neonatal MyHC in rat we compared My32 staining with an anti-neonatal MyHC antibody (clone WB-MHCn) which detects a weak band corresponding to neonatal MyHC and coincident with FMyHC 2b. We conclude that the predominant FMyHC forms detected by My32 in mouse embryos are therefore Adult FMyHC 2a, 2x and 2b. WB-MHCn weakly cross-reacts with the adult fast myosin marker (track M).

Embryonic skeletal muscles containing differentiated myotubes are first evident in mouse from around E11.5 (Fig. 1A–D). At E11.5 embryonic myotubes can be readily distinguished by their immunoreactivity to the pan-myosin antibody MF20 (Fig. 1A, E), whilst large numbers of Pax 7 staining skeletal muscle precursor cells delineate these newly forming muscle groups (Fig. 1B, F). Fast-myosin (FMyHC) is first detected by My32 antibody in a small number of secondary myotubes at E11.5 although Igf-2 cannot be detected in skeletal muscle myotubes at this stage (Fig. 1C–D, G–H). When proteins are separated on 7.5% acrylamide gels all three FMyHC isoforms (type 2a, 2x and 2b) are detected, low levels of neonatal myosin are also present, running coincident with the 2a band, but which do not increase with embryonic stage suggesting that the predominant isoforms detected by My32 from E12.5 are the three adult FMyHC (Fig. 2(C–D)). Whole embryo immunoblotting shows a rapid increase in the amount of FMyHC present in mouse embryos between E11.5 and E17.5 (Fig. 1I). This is reflected in the immunostaining pattern for fast myosin between E13.5 to E15.5 where there is a large and rapid increase in the number of FMyHC positive secondary myotubes in all embryonic muscle groups during these stages. Figure 1(J–Z) illustrates the dynamic staining pattern of fast myosin and Igf-2 in muscles of the forelimb at E13.5, E15.5, E17.5 and just after birth (P1).

In skeletal muscle, Igf-2 is first detected in a small number of myotubes at E13.5 in some, but not all, muscle groups (Fig. 1J–L). Specificity of the Igf-2 antibody is shown in Fig. 2(A–B). At this stage the localisation of fast MyHC is considerably more extensive than that of Igf-2 with a majority of secondary myotubes staining positively for fast myosin (Fig. 1K–M). Igf-2 positive myotubes at this stage were predominantly secondary myotubes which also contained fast myosin (Fig. 1L, M indicated by arrows). By E14.5 FMyHC is widely localised to secondary myotubes throughout the skeletal musculature of the embryo including in the muscles of the facial and distal limb which differentiate later than the central core and proximal musculature. From E14.5 the number of double positive (Igf-2 and fast-myosin) myotubes increases substantially in embryonic muscles, as does the intensity of staining for both proteins (data not shown). By E15.5 both FMyHC and Igf-2 are widely localised to secondary myotubes throughout the embryonic musculature and this pattern is largely coincident with the mRNA expression pattern in skeletal muscle at the same stage (Fig. 1N–P). Careful matching of adjacent sections stained respectively with Igf-2 and fast MyHC demonstrates that the Igf-2 staining pattern is largely coincident in secondary myotubes with that for fast myosin at E15.5 (Fig. 1Q–R, indicated by arrows). Thus by E15.5 just under half of all myotubes stain positively for fast myosin (47 ± 2.5 for diaphragm) and a majority (>90%) of these also stain positively for Igf-2 (44 ± 0.3 for diaphragm). Both primary and secondary myotubes containing Igf-2 and FMyHC are seen at this stage (Fig. 1Q, R, indicated by arrows). At E15.5 a majority of embryonic skeletal muscles contain FMyHC and most of these also contain comparable numbers of Igf-2 positive myotubes. A small number of muscles contain FMyHC but very little Igf-2 staining (Fig. 1O–P, indicated by arrows), these may be muscles destined to become predominantly slow (oxidative) muscles. Very rarely (<1% of myotubes) Igf-2 but not FMyHC staining was seen. The proportion of FMyHC positive fibres in any particular muscle group appears to be stable (see also Fig. 5). By E17.5 there is a sharp decline in the intensity of Igf-2 staining in the majority of embryonic myotubes and an increased heterogeneity in staining intensity of fast myosin positive fibres (Fig. 1S–V). Whole embryo immunoblotting demonstrates a substantial increase in staining for Igf-2 at E13.5 and E15.5 which is reduced at E17.5 reflecting this dynamic staining pattern for Igf-2 in skeletal muscle myotubes (Figure 1I). Igf-2 is also present in non-muscle tissues (including liver, kidney, heart, gut) between E11.5 and E17.5 (Figure 1I; Figure 2A–B). By P1 (day one post birth) both fast-myosin and Igf-2 are absent from a majority of muscle fibres (Fig. 1W–Z). Thus both Igf-2 and fast myosin display a dynamic staining pattern during secondary myogenesis in which both proteins are widely localised to secondary myotubes peaking in intensity around E15.5. The Igf-2 pattern is more narrowly defined, beginning later (E13.5) and declining earlier (E17.5), than that of FMyHC suggesting that Igf-2 plays a specific role during the growth (myotube expansion) and maturation phases of late secondary myogenesis rather than in the initiation of secondary myotubes..

### Forced over-expression of Igf-2 in myotubes induces the generation of fast twitch muscle fibres

**Figure 3:**
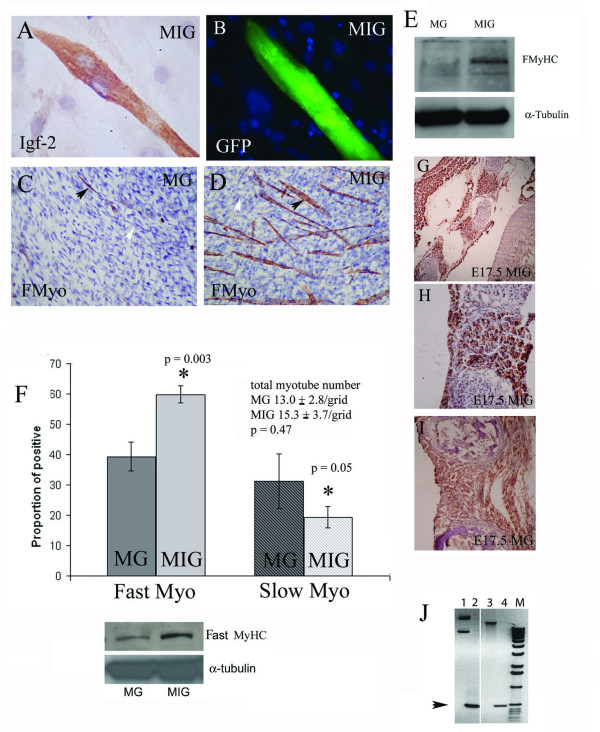
Differentiated C2MIG myotubes, but not undifferentiated myoblasts, show (A) strong immunostaining (brown) for IGF-2, (Counterstain is Haematoxylin (blue)) and (B) bright green fluorescence (GFP). DAPI counterstained nuclei (blue) reveal that undifferentiated cells do not express the GFP transgene. Spontaneous differentiation into fast MyHC positive myotubes occurred (C) rarely in C2MG (B9) cells but more frequently (D) in C2MIG (H19) cells cultured under non-permissive conditions (DMF12 and 10% FCS) for 5 days. A majority of C2MG (B9) and a minority of C2MIG (H19) myotubes formed are fast MyHC antibody negative (C-D; white arrows). A minority of C2MG myotubes and a majority of C2MIG myotubes, are FMyHC positive (C-D; Black arrows). Total myotube numbers: MG, 16.4 ± 3.2; MIG 19.1 ± 3.5 ; Students t-test, p = 0.76. The average number of nuclei (per myotube) was 5.48 ± 1.17 (s.d.). (E) Immunoblotting shows a substantial increase in FMyHC in C2MIG compared to C2MG under these conditions. (F) After 3 days in differentiation permissive media there was a 50% increase (*, p = 0.003) in the proportion of FMyHC positive myotubes in C2MIG (H19) cells compared to C2MG (B9) and a concomitant 30% reduction (p = 0.05) in the proportion of SMyHC positive myotubes in C2MIG (H19) compared to C2MG (B9). Total myotube number was not altered (p = 0.47). Graph shows the mean and standard deviation of 3 separate experiments, each performed in duplicate. Immunoblot shows the increase in FMyHC in C2MIG compared to C2MG under the same regime. (G) E17.5 MIG embryos show a very strong generalised up-regulation of FMyHC in all skeletal muscles. (H) In MIG intercostals muscles almost all myotubes have intense FMyHC staining compared to (I) MG embryos at E17.5. (J) A probe recognising the intersection between the pmCK and Igf-2 was used to genotype MIG transgenic positive (track 4) and MIG transgene negative (track 3) mice. Track 1, MG and track 2, MIG plasmid PCR, demonstrate the specificity of the MIG PCR primers. M - 1Kb ladder. PCR to genotype MIG transgene positive (track 4) and MIG transgene negative (track 3) mice. Primer set mCKIgf-2 1. Data shown is of one MIG+ and one MG+ E17.5 embryo. The phenotype is representative of 3 other E17.5 MIG+ embryos immunostained for FMyHC. 2 MG+ E17.5 embryos and 4 E17.5 MIG- littermates showed an identical FMyHC phenotype to WT C57BL10 E17.5 embryos (Figure 3I and Figure 1).

To further establish the role of Igf-2 in fast twitch muscle fibre formation we generated clonal skeletal muscle cell lines which specifically over-express Igf-2 in differentiated myotubes. To achieve this we used a bicistronic transgene (MIG) which conditionally drives the independent expression of Igf-2 and GFP in differentiated myotubes under the control of the full length muscle creatine kinase promoter (p3300mCK). The p3300mCK is activated during myotube differentiation and is expressed in all fully differentiated myotubes but not in undifferentiated myoblasts [28]. Clonally derived, MG, cells which express GFP but not Igf-2 under the control of the pmCK were used as a control. Cell lines were generated by transfecting MG or MIG respectively into C2C12 cells followed by clonal derivation of picked colonies by serial dilution. Plating efficiency and colony formation assays carried out on C2MG (B9) and C2MIG (H19) cells showed no significant difference between the two lines (Student t test: p = 0.23 and p = 0.60 respectively). Consistent with the reported expression pattern, neither GFP nor IGF-2 is present in proliferating, undifferentiated C2MIG cells whilst C2MIG myotubes express both proteins (Fig. 3A–B). The timing of GFP expression in C2MG cells is comparable to that of C2MIG (not shown). In high density cultures, C2MG (and other wild-type) myoblast cell lines, undergo sporadic spontaneous differentiation to form myotubes but only a minority of these myotubes express fast MyHC (black arrows, white arrows indicate FMyHC negative myotubes) (Fig. 3C). Spontaneously differentiating high density cultures of C2MIG expressing cells on the other hand, have a substantially increased proportion of FMyHC positive myotubes and increased FMyHC protein suggesting that the presence of Igf-2 in these myotubes increases the likelihood of fast MyHC expression in the resultant myotubes (Fig. 3D–E).

When C2MIG and C2MG cells are placed at lower culture densities under differentiation permissive conditions (low serum, 2% horse serum) both will differentiate extensively after 3 days. Whilst there is no difference between the total number of myotubes generated by the two cell lines after 3 days there is a striking (and statistically highly significant) difference in the proportion of fast and slow MyHC positive myotubes generated by the two cell lines (Fig. 3F). C2MIG expressing cells consistently generate 30% more fast MyHC positive myotubes (59.8 ± 2.9 %) than do C2MG (39.3 ± 4.8 %) and a 30% reduction in the proportion of slow (Type 1) MyHC positive fibres (Fig. 3F). Western blotting confirms the increased expression of fast MyHC in differentiated C2MIG compared to C2MG myoblasts (Fig. 3F). These data clearly demonstrate that over-expression of Igf-2 in mouse skeletal myotubes promotes fast MyHC positive myotube formation; the concomitant reduction in slow MyHC positive fibres suggests that it may induce some myotubes to switch from slow to fast MyHC.

**Figure 4:**
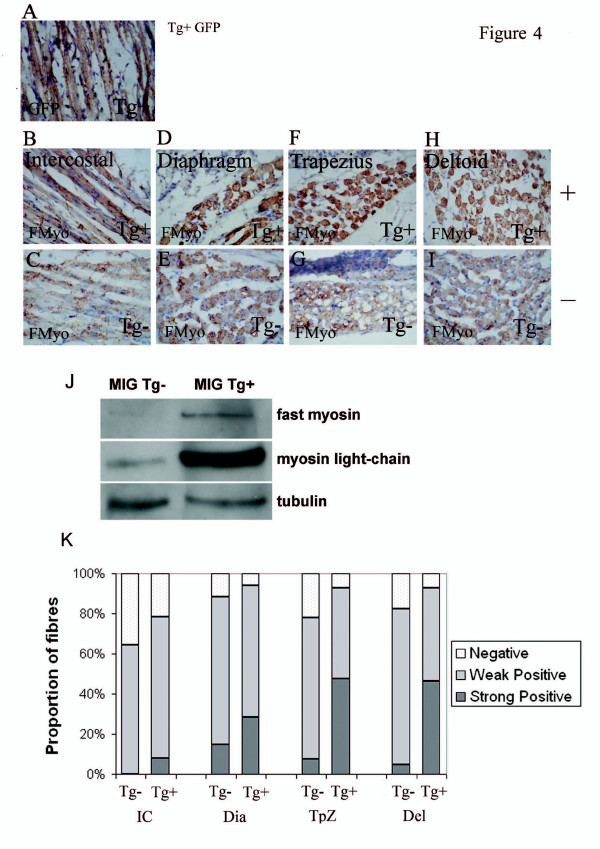
One day old pups (P1) carrying the MIG transgene exhibit, (A) GFP immunoreactivity in all of their skeletal muscle fibres and show up-regulation of fast-myosin in (B) intercostal, (D) Diaphramatic, (F) Trapezial and (H) Deltoidal muscles compared to (C, E, G, I) the same skeletal muscles in their non-transgenic litter mates. (J) Immunostaining demonstrates that fast-myosin heavy chain (fast MyHC) is barely detectable at P1 in non-transgenic pups. Both fast MyHC and fast myosin light chain (MyLC) are substantially up-regulated in P1 MIG transgenic mice. (K) Comparison of the number of muscle fibres positively and negatively staining for fast-myosin shows an increase in the proportions of fast-myosin positive fibres in MIG transgenic muscles and a substantial shift from weak to strong staining in intercostal (IC), diaphragm (Dia), Trapezius (TpZ) and Deltoid (Del) muscles compared to non-transgenic controls. Data shown is of one MIG+ P1 pup and a non-transgenic control. Three other pups analysed in the same way showed essentially the same phenotype. 2 MG P1 pups had a phenotype indistinguishable from the WT littermate. GFP (and Igf-2) immunostaining patterns in both MG and MIG were consistent with published expression patterns for mCK.

Mice carrying the MG transgene display strong GFP expression in their skeletal muscle myotubes at E17.5 and P1 and like wild type, contain very little Igf-2 in their myotubes at E17.5 and during early postnatal life. MIG mice on the other hand, exhibit labelling of both Igf-2 and GFP in their myotubes in late gestation (E17.5) and the early postnatal period (Fig. 4A). Immunostaining for FMyHC demonstrates a substantial increase in staining intensity and an excess of FMyHC positive secondary myotubes in the skeletal muscles of MIG at E17.5 which corresponds with the increased levels of Igf-2 in the myotubes of these embryos (Fig. 3G–H). MG embryos at this stage have a level of FMyHC staining comparable to that seen in wild type (Fig. 3I). The characterisation of MIG positive embryos is shown in Fig. 3J. These data strongly support a role for Igf-2 in the maintenance and regulation of FMyHC in embryonic myotubes.

**Figure 5:**
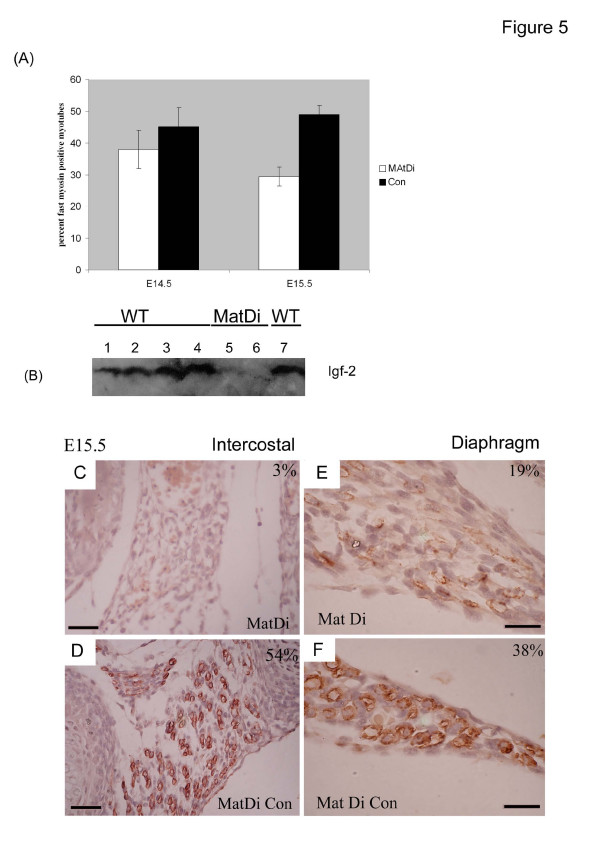
(A) The proportion of fast MyHC myotubes is decreased in Igf-2 deficient mice (maternal disomy Chromosome 7 - MatDi) at E14.5 compared to WT sibling controls (Student t-test, p = 0.06). By E15.5 there is an overall drop of 50% in the proportion of fast MyHC in MatDi embryos compared to WT (statistically significant by Student t-test, p = 0.03). (B) Embryo immunoblotting for Igf-2 demonstrates a stage related increase in Igf-2 protein between E10.5 and E15.5 in WT and absence of Igf-2 in MatDi embryos at E14.5 and E15.5 Tracks 1–4 E10.5, E13.5, E14.5 and E15.5 WT embryos respectively, Track 5 E14.5 MatDi, Track 6 E15.5 MatDi and Track 7 E15.5 WT. (C) In some Mat Di muscles fast MyHC staining is almost entirely abolished compared (D) to WT siblings in which over half of myotubes at this stage contain fast MyHC. In the posterior insertional region of the diaphragm, the intensity of staining and number of fast MyHC positive skeletal myotubes at E15.5 is, (E) decreased in maternal disomy mutant embryos (19% fast MyHC positive myotubes) compared to (F) litter mate controls (MatDi Con 38% fast MyHC myotubes). Magnification bars represent 10 microns.

### Over-expression of Igf-2 prevents down-regulation of fast-myosin at birth in MIG+ mice

The down-regulation of fast-myosin in the skeletal muscles of neonates is thought to be related to the process of establishing mature skeletal muscle groups which is completed (in mice) during the first 1–2 weeks post birth. The close correlation during myogenesis between the localisation patterns of Igf-2 and fast skeletal myosin (Fig. 1) together with the excess production of fast MyHC containing myotubes when Igf-2 is over-expressed (Fig. 3) suggest a role for Igf-2 in regulating this process. 4 MIG positive pups were stained for FMyHC and all had a substantial increase in the proportion of neonatal muscle fibres containing fast MyHC compared to non-transgenic sibling controls. The complete analysis of one representative MIG+ pup is shown in (Figure 4B–I) compared to a wild type sibling control which was processed in parallel (Fig. 1W–Z). Mice carrying the MIG transgene strongly express both Igf-2 and Gfp in their skeletal muscles on day 1 of birth (Fig. 4A). These mice also exhibit strong and extensive FMyHC staining in all of their myofibres and throughout their skeletal musculature (Fig. 4B, D, F, H). Comparison with wild-type (non-transgenic) siblings showed an extensive down-regulation of fast MyHC protein in the same muscles (Fig. 4C, E, G, I). Protein from the hind part of this pup was subject to immunoblotting and confirms a substantial increase in fast myosin heavy and light chain proteins in MIG compared to sibling control muscles (Fig. 4J). Three other MIG+ pups also show an increase in the FMyHC band compared to non-transgenic littermate controls (not shown). Detailed analysis of individual muscles revealed that MIG muscles revealed both an increase in the intensity of fast-myosin staining compared to wild type as well as an increase in the proportion of fibres retaining fast-myosin (Fig. 4K). This difference is most striking in muscle groups such as the Deltoid and Trapezius destined to become mixed fibre muscles (Fig. 4F–I, K). A shift towards fast myosin positive fibres was also seen in the diaphragm and intercostal muscles (Fig. 4B–E, K).

### Loss of Igf-2 expression results in a substantial reduction in the proportion of fast-myosin positive myotubes in embryonic muscles

To establish a functional relationship between the generation of fast-twitch myotubes and Igf-2, we examined the number of fast MyHC positive fibres in the embryonic skeletal muscles of a parental origin disomy mutant which shows disruption of embryonic Igf-2 (and several genes which regulate Igf-2 expression [29]). Maternal chromosome 7 disomy (MatDi) mutant mice show total loss of their (paternal) monoallelic Igf-2 expression resulting in an absence of skeletal muscle Igf-2 expression as well as loss of circulating Igf-2 ([30]; Figure 5). We compared the number, intensity of staining and localisation of fast MyHC positive myotubes in MatDi mouse embryos at E14.5 and E15.5 to those of their wild-type sibling controls. At E14.5 the pattern of fast MyHc staining in MatDi embryos is generally comparable to wild type except that both the intensity of staining and the proportion of fast MyHC containing myotubes is reduced (Fig. 5A). Interestingly, FMyHC+ myotubes were not abolished despite the complete suppression of Igf-2 protein (Figure 5B). Counting the proportion of fast MyHC positive myotubes in a range of different muscles (distal and proximal limb, intercostal, diaphragm; trunk) reveals a consistent decrease in the number of fast MyHC positive myotubes in MatDi compared to WT at E14.5 and E15.5 (Fig. 5A; Paired t-test: E14.5, p = 0.0601; E14.5, p = 0.03). At E15.5 there is a greater reduction in the number of fast MyHC positive secondary myotubes over all muscles (compared to E14.5) and there is still considerable heterogeneity between muscle subgroups with the fast MyHC positive myotube component ranging from being almost entirely absent in some muscles (Fig. 5C), and up to half of wild type numbers of fast MyHC + myotubes in other muscles (Fig. 5E–F). In the respiratory muscles, FMyHC staining was almost absent in MatDi intercostals compared to the wild type in which around half of myotubes are FMyHC+ (Fig. 5C–D) whilst in the MatDi Diaphragm the number of fast MyHC positive myotubes was around half that of WT controls (Fig. 5E–F).

### ShRNA Knockdown of Igf-2 reduces the proportion of fast-myosin positive myotubes in differentiating muscle cultures

The data presented in the previous section showing a reduction but not complete abolition of FMyHC+ myotubes in the absence of Igf-2 (Fig. 5) show that Igf-2 is not required for myotube induction and suggest that Igf-2 may be essential for the maintenance and maturation of the fast twitch myotube component in embryonic muscles *in vivo *rather than for their *de novo *synthesis. To test this role for Igf-2 we used ShRNAi to knockdown Igf-2 in differentiating myoblasts.

**Figure 6:**
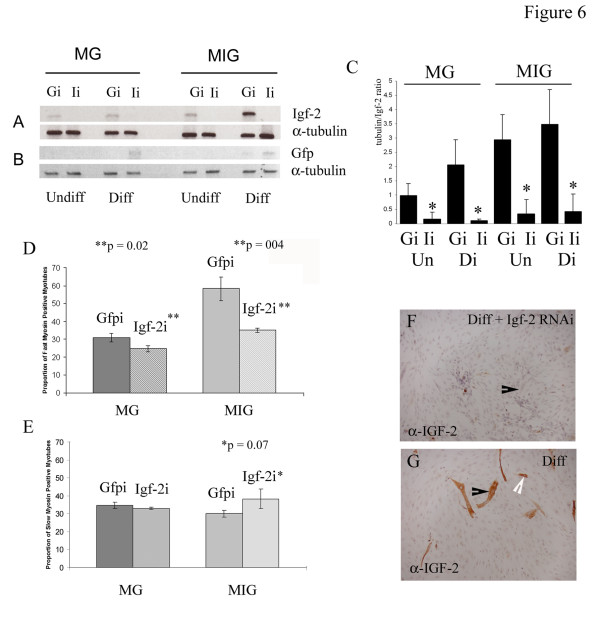
Semi-quantitative RT-PCR for (A) Igf-2 and (B) Gfp C2MG and C2MIG cells transfected with RNAi constructs respectively targeting Igf-2 (pShagIgf-2, Ii) and Gfp (pShagGfp, Gi). (C) Densitometry of Igf-2 signal expressed as a ratio of α-tubulin controls confirms that pShagIgf-2 (Ii) but not pShagGfp (Gi) abolishes the Igf-2 message (* statistically significant, p < 0.05, Student t-test). (A, C) Endogenous Igf-2 mRNA is detected at very low levels in undifferentiated (*Un*) wild-type (C2MG) and C2MIG cells and is upregulated following differentiation (*Di*). Substantial induction of ectopic Igf-2 mRNA is seen in C2MIG expressing cells following differentiation. pShagIgf-2 abolishes both endogenous and ectopic (C2MIG) Igf-2 mRNAs. (B, C) Gfp mRNA is not present in undifferentiated C2MG or C2MIG cells but is detected in both cell lines following differentiation. pShagGfp abolishes Gfp mRNA in these cells. (D) C2MIG cells transfected with pShagIgf-2 show a substantial reduction in the proportion of their fast-myosin positive myotubes which is statistically significant (Student t-test, p = 0.004, indicated by **) and, (D) a concomitant increase in the proportion of slow myosin positive myotubes (statistically significant, P = 0.07 *). Abolishing endogenous Igf-2 expression in C2MG cells results in, (D) a smaller but statistically significant (p = 0.02, **) drop in the number of fast-myosin positive myotubes but (D) does not increase the number of slow-myosin positive myotubes. Abolishing Gfp using pShagGfp has no effect on the proportion of (D) FMyHC or (D) SMyHC positive myotubes present in either C2MG or C2MIG cells. Igf-2 RNAi abolishes Igf-2 immunostaining in (F) C2MIG myotubes. (G) Igf-2 is strongly expressed in differentiated C2MIG myotubes (F-G; black arrows indicate myotubes). (G) Igf-2 is present in small numbers of mono-nuclear bipolar cells consistent with the reported expression of muscle Creatine Kinase (mCK) just prior to differentiation (white arrow). Total myotube number for cells treated with lipofectamine, pShagGfp and pShagIgf-2 was analysed by ANOVA and showed that the number of myotubes generated was not affected by the treatment regime, p = 0.64 (MG), p = 0.23 (MIG).

We used a shuttle vector (pShag1) to generate ShRNA constructs targeting GFP and Igf-2 (pShagGfp, pShagIgf-2 respectively) which knockdown their respective targets with high efficiency. For details of these vectors see the methodology section and [31]. Semi-quantitative RT-PCR for Igf-2 shows that C2MG myoblasts express low levels of endogenous Igf-2, similar to levels expressed by undifferentiated C2MIG cells (Fig. 6A). As expected Igf-2 expression levels are strongly up-regulated in C2MIG cells during differentiation as the MIG transgene is switched on. Weak induction of Igf-2 in differentiated C2MG cells is due to the presence of small numbers of Igf-2 positive myotubes in these cells. Using semi-quantitative RT-PCR for Gfp we show that Gfp message is detectable only in differentiated C2MG and C2MIG cells and is abolished in cells transfected with pShagGfp but not those expressing pShagIgf-2 (Fig. 6B). Densitometry analysis demonstrates that both endogenous and MIG generated Igf-2 message are effectively suppressed in cells transfected with pShagIgf-2 demonstrating ShRNA knockdown close to 100%. Igf-2 message is not affected in cells transfected with an RNAi construct targeting Gfp (pShagGfp) (Fig. 6A, C). ShRNA knockdown of Igf-2 abolished the shift towards fast-myosin myotube production seen in C2MIG cells (Fig. 6D). C2MIG cells transfected with pShagIgf-2 display a 50% decrease in the proportion of fast myosin positive myotubes and a 30% increase in the proportion of slow myosin positive myotubes compared to pShagGfp (or control C2MIG) transfected myotubes, suggesting that a majority of the excess fast myotubes generated by C2MIG cells (shown in Fig. 3) are made at the expense of generating slow myosin containing myotubes (Fig. 6D–E). ShRNA knockdown of endogenous Igf-2 in C2MG cells resulted in a 15–20% reduction in the proportion of FMyHC+ myotubes compared to control C2MG or pShagGfp transfected C2MG cells demonstrating that endogenous Igf-2 plays a role in the generation of fast myotubes (Fig. 6D). Interestingly, there was no statistically significant effect on the proportion of slow-myosin positive fibres present in these cultures suggesting that loss of endogenous Igf-2 expression alone is insufficient for the promotion of slow twitch myotubes under WT conditions. Loss of Igf-2 protein, in Igf-2 ShRNA treated C2MIG cells, is shown in Fig. 6F. In these cultures myotubes are formed but do not express Igf-2 (Fig. 6F, black arrow).

The timing of mCK expression in C2MIG cells coincides with the appearance of endogenous Igf-2 in wild-type skeletal muscle myoblasts (Fig. 6G). As in wild-type C2C12 (and C2MG) cells Igf-2 protein is not present in proliferating cells but can be first detected in a small number of cells with bipolar morphology around 24 hours after switching them into differentiation permissive medium (2% horse serum) (white arrow Fig. 6G). By 3 days in differentiation medium fully formed multinucleate, Igf-2 positive myotubes can also be identified (Fig. 6G, black arrow). Interestingly in clonally derived WT myoblast cultures, only about half of myotubes formed in culture under differentiating conditions contain Igf-2 and Igf-2 negative myotubes can also be identified. The presence of a mixed population of Igf-2 positive and negative myotubes, models that seen in the embryo during late secondary myogenesis (Fig. 1).

## Discussion

In this study we show for the first time that Igf-2 protein localises to skeletal muscle myotubes during secondary myogenesis and that it is specifically and progressively associated with the fast-myosin positive secondary myotube population during the late growth and maturation phases of embryonic myogenesis. The Igf-2 localisation pattern largely coincides with the previously reported mRNA expression pattern for this growth factor [21,23,24], but the increased resolution afforded by immunostaining demonstrates the localisation of Igf-2 to embryonic muscle more precisely and shows that it is restricted to a subset of myotubes during secondary myogenesis. The close correlation between the protein and mRNA expression patterns suggests that this growth factor acts in a predominantly cell autonomous (autocrine) manner in these myotubes. The Igf-2R, a negative regulator of secreted Igf-2, which plays an important role in regulating embryonic levels of systemic Igf-2 is extensively expressed in embryonic skeletal muscle, and thus may contribute to the regulation of this process [32].

### Igf-2 localisation specifies the fast twitch motor unit during secondary myogenesis

Igf-2 protein localises to embryonic muscle myotubes later (E13.5) than FMyHC (E11.5) and is also down-regulated earlier (E17.5 compared to P1 for FMyHC) precluding a role for Igf-2 in the initiation of secondary myotube differentiation. This conclusion is supported by the finding that in Igf-2 deficient (MatDi) embryos, whilst the FMyHC+ myotube population is diminished, it is not absent and myogenesis is not impaired. The localisation pattern of Igf-2 in embryonic myotubes during late secondary myogenesis correlates well with the transition from developmental to mature myosin expression, the so-called 'late transition window' described by several authors [6]. Its close correlation with FMyHC expression suggests a particular role for Igf-2 in establishing the fast twitch myotube component of these muscles. Like FMyHC, Igf-2 is predominantly found in secondary myotubes in the early stages of secondary myogenesis although it shows a more restricted pattern than does FMyHC. By late myogenesis (E15.5) Igf-2, like FMyHC, is localised to ~50% of embryonic myotubes and there is a striking coincidence in the localisation patterns of the two proteins, both of which are found in (presumptive fast twitch) motor units (grouped primary and secondary fibres) which in a majority of muscles are evenly interspaced with FMyHC and Igf-2 negative motor units. This pattern is widespread (though not uniform) throughout the hypaxial and epaxial musculature but is particularly striking in fast and mixed-fibre embryonic muscle groups such as the Deltoid, Intercostals and Diaphragm. By E17.5 the intensity of FMyHC staining in WT embryonic muscle is heterogeneous with stronger staining being found in Igf-2 positive myotubes and staining intensity varies considerably from muscle to muscle. The mixed pattern of Igf-2 positive and negative myotube clusters present in WT embryos from E15.5 seems to be required to maintain this heterogeneity of staining intensity since when Igf-2 is expressed in all embryonic myotubes (as in MIG embryos) this variation is lost and FMyHC staining is generally uniformly intense in all muscles and in individual myotubes. When Igf-2 is lost (as in MatDi embryos) the converse is true and FMyHC staining is weaker throughout the entire musculature.

### Disruption of Igf-2 expression causes a shift in the FMyHC+ myotube population

In Igf-2 deficient embryos (MatDi) the proportion of FMyHC+ myotubes in embryonic muscles is halved at E15.5 although secondary myogenesis is not impaired. The presence of more weakly staining fast MyHC fibres in MatDi embryos at E14.5 and the subsequent decline in the proportion of FMyHC+ myotubes in MatDi embryos at E15.5 supports our conclusion from the immunostaining pattern of Igf-2 that Igf-2 is not necessary for the formation of fast secondary myotubes but supports the hypothesis that Igf-2 is required for the continued maintenance of FMyHC+ myotubes or conversion of subsequent myotubes into fast-twitch fibres. This argument is also supported by the finding that when Igf-2 is over-expressed (in MIG mice) the proportion of FMyHC myotubes is increased such that by E17.5 almost 100% of MIG myotubes contain strong FMyHC staining. Expression of MIG *in vivo *causes a substantial shift in the proportion of FMyHC+ myotubes in embryonic muscles as well as in the intensity of staining suggesting that Igf-2 may directly induce FMyHC. Thus by manipulating Igf-2 expression in late gestation secondary myotubes it is possible to substantially alter both the proportion and intensity of expression of FMyHC in embryonic myotubes *in vivo*.

In addition to Igf-2, there are a number of imprinted genes contained within the duplicated region of the distal tip of chromosome 7 in MatDi mice [29]. A majority of these are not imprinted and/or not expressed in embryonic skeletal muscles. *Mash-2 *and *Ins-2 *for example, are imprinted only in extraembryonic tissues and play roles in placental function, they are unlikely to influence the reduced FMyHC+ myotube phenotype described here. In addition to Igf-2, two genes (*H19, cdkn1c*) are known to express in skeletal muscle, both are maternally imprinted and thus up-regulated in MatDi embryos. The net effect of maternal duplication in embryonic skeletal muscle is therefore the complete suppression of Igf-2 and its downstream effect. H19 is a non-translated mRNA which negatively regulates Igf-2 at the mRNA level and is unlikely to influence the muscle phenotype of these mice other than through the suppression of Igf-2 expression. The cyclin-dependent kinase inhibitor, *cdkn1 *(p57^kip2^) is negatively regulated by Igf-2 [33]. It is expressed in skeletal muscle and so may mediate the Igf-2 signal in this tissue [33,34]. Mice lacking both Cdkn1 and p21, but not cdkn1 alone, have a muscle phenotype and cdkn1 may thus have a role in myotube differentiation, over-expression of cdkn1 (as in MatDi) is not however, reported to affect muscle function [35]. Taken together with our remaining data, the suppression of FMyHC myotubes in MatDi muscles is most likely attributable to the absence of Igf-2 rather than to the over-expression of Cdkn1.

By forcing Igf-2 expression into more myotubes *in vitro*, we alter the proportion of FMyHC positive myotubes generated by cultured myoblasts. In vivo, forcing Igf-2 expression into all myotubes both increases the proportion of FMyHC positive fibres and abolishes the heterogeneity of FMyHC staining which is established in late gestation (E17.5) embryos. Igf-2 therefore appears to be necessary for controlling the number of fast myotubes formed throughout the musculature and may play a crucial role in the mechanism of fibre-type switching which establishes the correct proportion of fast and slow twitch fibres in each individual muscle group.

### Igf-2 regulates the post-natal FMyHC pattern

Both Igf-2 and fast-myosin are previously reported to substantially down-regulate in mice at birth [6,36]. Our data demonstrates that Igf-2 and FMyHC are sequentially suppressed at the end of myogenesis with Igf-2 downregulation preceding that of FMyHC. By E17.5, in wild type (and MG) embryos, Igf-2 is almost entirely absent from embryonic muscle and FMyHC staining is less intense in some myotubes (compared to E15.5). By birth, consistent with previous reports, both proteins are strongly suppressed. MIG induces a generalised increase in the intensity of fast MyHC staining and in the number of fast MyHC positive myotubes in embryonic skeletal muscles. MIG causes a very striking elevation of fast MyHC staining in embryonic myotubes at E17.5 in comparison to MG. When Igf-2 is prevented from down-regulation at E17.5 (as in MIG embryos) FMyHC also fails to down-regulate, resulting in a substantial increase in FMyHC+ myotubes at E17.5 and a very substantial excess of FMyHC+ muscle fibres in P1 neonates. These data strongly indicate a role for Igf-2 in the specification of the FMyHC+ myotube.

In vertebrates a focus of recent research has been the role of innervation in determining skeletal muscle fibre type post-natally. Depending on species and the skeletal muscle examined, experimental deinnervation of adult mammalian skeletal muscles has been shown to cause a shift in fibre type expression from fast to slow or from slow to fast myosin fibre types [8,37]. It is also possible to modulate slow and fast myosin expression patterns in post-natal muscles by loading and unloading muscles by means of exercise or forced inactivity respectively [38]. In some of these studies growth factor expression changes have been reported and both Calcineurin and NFATc3 signalling have been shown to mediate fibre switching (towards slow MyHC) in response to neural and loading cues suggesting that growth factor signalling may mediate post-natal switches in myosin type induced both by innervation and mechanical load [39-41]. Igf-2 is the first growth factor which promotes fast myosin expression in embryonic muscle fibres.

Igf-1, a growth factor which is closely related to Igf-2 but with distinct function and regulatory pathways in embryogenesis, induces a very substantial myotube hypertrophy when over-expressed in embryonic myotubes [42]. Igf-2 does not appear to induce this same effect and we found no evidence of hypertrophy in MIG embryos, neonates or cultured MIG myotubes. Similarly, in neither MatDi nor Igf-2 ShRNA transfected myotubes did we see a reduction in the diameter of myotubes compared to WT or untreated myotubes. This is consistent with a large body of data which suggest that the two growth factors have distinct roles in embryogenesis [13,22,24,43]. Detailed analysis of the respective roles of Igf-1 and Igf-2 in the embryonic nervous system and preimplantation development support this view and suggest that Igf-2 signalling may require the Igf type 2R (Igf-2R) in early embryogenesis [22,44]. Unlike Igf-2, Igf-1 is not expressed in embryonic skeletal muscle, although it is present in non-muscle mesenchymal cells and embryonic neural tissues. The Igf type 1 receptor (type 1R) is more widely expressed and can mediate signal of both peptides in many tissues, however data from phenotypes of Igf axis null embryos suggests that both function and signalling of the two peptides in the embryo is distinct. This is especially true in late embryogenesis (coincident with secondary myogenesis) when Igf-2 function does not depend on the type 1R but instead acts via alternative receptors such as the Insulin receptor (InsR) or hybrid receptors [24,43,45,46]. Igf-2 function is also extensively regulated by the Igf-2R [32,47]. Both Igf-2 and Igf-2R expression are regulated by genomic imprinting.

### Medical implications

The regulation of fibre-type is of importance to the elucidation and treatment of Duchenne skeletal muscle dystrophy which shows preferential destruction of type 2 fibres resulting in the rapid loss of function of fast and mixed fibre muscles of the limb and trunk and in destruction of the fast-fibre component of the respiratory (intercostals and diaphragmatic) muscles [48]. It is these muscles in particular in which we report altered Igf-2 expression to have its most substantial effect on the embryonic and perinatal fast twitch myotube population. Detailed analysis of individual muscles in MIG+ mice revealed both an increase in the intensity of fast-myosin staining compared to wild type as well as an increase in the proportion of fibres retaining fast-myosin. Whilst this difference was most striking in muscle groups such as the Deltoid and Trapezius which are destined to become mixed fibre muscles, there was also a shift towards FMyHC positive fibres in the diaphragm and intercostal muscles. In the human and rodent adult these specialised respiratory muscles have a finely balanced composition which ranges between 40–50% Type 1 (slow myosin) and 50–60% Type 2 (fast myosin) which is essential for their role in respiration [49,50]. Whilst MIG mice do not have respiratory difficulties at birth, the increase of Type 2 myosin in their respiratory muscles may have long term consequences on the stamina or adaptability of their diaphragmatic and intercostal muscles.

Some growth factors including Igf-2 and growth hormone have been demonstrated to exert an ameliorative effect on the juvenile dystrophic phenotype in the *mdx *mouse (a genetic model for DMD). This suggests that dystrophic fast-twitch muscle fibres could be particularly vulnerable to the rapid down regulation of Igf-2 which takes place at birth in both mouse and human and may benefit from supplementary post-natal Igf-2, providing an important therapeutic approach for this severe and intractable disease [27,51]. In DMD, children usually die from respiratory failure associated with loss of function of the respiratory muscles. Growth hormone (GH) has been shown to improve tensile strength in *mdx *diaphragm, whilst Igf-1 inhibits myofibre breakdown in mixed fibre muscles such as the quadriceps although not in the diaphragm [51,52]. Here we show that it is possible to regulate the production of fast twitch fibres by manipulating the expression of Igf-2 in vivo or in cultured myotubes. The respiratory muscles appear to be particularly sensitive to Igf-2 expression levels and Igf-2 promotes fast fibre production in these muscles in both embryonic and post-natal intercostal and diaphragmatic muscles suggesting that Igf-2 may be particularly useful in therapeutic strategies targeting the respiratory muscles.

## Conclusion

Igf-2 localises to ~50% of myotubes during a narrow time window in late secondary myogenesis coinciding with embryonic fibre-type transition, and is required for establishing the correct proportion of fast twitch motor units in embryonic and neonatal muscle groups.

## Methods

### Preparation of mouse embryo samples

Mouse pairs were established as natural matings to generate embryonic stages E10.5 to E17.5 (morning of plug detection counted as E0.5). Pregnant females were culled by cervical dislocation (in accordance to Schedule I method of the Animal (Scientific Procedures) Act 1986). Maternal disomy chromosome 7 (MatDi) embryos and their wild type (WT) litter mate siblings (obtained from Anne Ferguson Smith, University of Cambridge, UK) were produced by intercrossing mice carrying stable robertsonian translocations as described previously in [34,53]. Embryos from mixed MatDi and WT litters were fixed and subsequently stained in parallel. All embryos were fixed in 4% paraformaldehyde (PFA) overnight, followed by subsequent dehydration and embedding in paraffin wax as previously described [27,34]. All sections were sagital (5μm).

### Immunohistochemical (IHC) detection of Igf-2 and myosin subtypes

**Embryo and P1 pup sections **were de-waxed and dehydrated before being subjected to an appropriate antigen retrieval method (Phem Triton for 2 minutes or 2 minutes high pressure immersion in hot sodium citrate buffer; pH 6.0), as described previously [34]. Endogenous peroxidase was blocked using hydrogen peroxide, (3% H_2_O_2 _in water and 0.5% H_2_O_2 _in Methanol), slides washed and then blocked in 3% BSA in phosphate buffered saline (PBS) for anti-Igf-2 or 3% Milk in PBS (all myosin antibodies), or TNB buffer (for tyramide signal amplification). Adjacent sections were incubated overnight at 4°C in primary antibodies; anti-Igf-2 mouse monoclonal IgG1 (1/100, Upstate Biotechnologies); anti-skeletal myosin (fast) IgG1 (1/1000, Sigma, Clone MY32); anti-pan myosin (1/1000, MF20); anti-slow myosin mouse monoclonal (1/100, A4.840) and anti-Pax 7 (1/500) all from Developmental Hybridoma Studies Bank (DHSB), Iowa City); anti-GFP (1/1000, MBL); antibody concentrations established by titration) or (secondary antibody control) in diluent (1% blocking buffer) alone. Secondary goat-anti-mouse biotinylated antibody (1/1000 in 1% blocking buffer, Amersham Life Sciences) was incubated at 20°C for 1 hour. Visualisation was achieved using either vector ELITE ABC detection (Vector Laboratories Ltd) or tyramide signal amplification (TSA: PerkinElmer Life Sciences) followed by 1mg/ml 3,3' diaminobenzidine tetrahydrochloride (DAB Dako Ltd). Embryos were counterstained with Gill's Haematoxylin (BDH, UK) using standard protocols. Where sections are compared in figures the same protocol was used for each section and antibody (see figure legends). Cell lines were immunostained using the same protocols (analysis is described below).

### Antibody specificity

The specificity of the Igf-2 primary antibody (upstate Biotechnologies) was established by pre-incubation of the antibody with 2g/ml Igf-2 (Growpep) for 1 hour at room temperature. Pre-absorbed antibody was used in the immunstaining procedure as described above. Pre-absorption of the anti-Igf-2 antibody with 2μg/ml Igf-2 peptide completely abolished Igf-2 immunostaining in all regions where signal was obtained, shown here for both embryonic liver and diaphragm (Fig. 2 A-B). The pattern of immunolocalisation of Igf-2 broadly followed that published for mRNA in situ [24,54,55,36] and showed Igf-2 to be distributed widely throughout the developing embryo in a range of tissues including omphalocoele, skeletal muscle, brain, liver, kidney, heart, gut and developing bone (to be published elsewhere). During the second half of gestation a predominant site of Igf-2 localisation is developing skeletal muscle (see Figure 2). The antibody used to detect fast myosin isoforms in this study is a monoclonal antibody My32. This antibody is reported to detect all three fast myosin isoforms (2a, 2b and 2x/d) but not recognise developmental or cardiac myosin isoforms. Cross-reactivity with neonatal MyHC has been reported for this antibody in rat [56] although neonatal myosin was not formally identified in this study. To establish the specificity of the anti-FMyHC (clone My32) antibody whole embryo proteins were electrophoresed in duplicate on 7.5% SDS-PAGE to allow separation of myosin isoforms and immunoblotted for FMyHC and Neonatal MyHC (WB-MHCn) (half the blot for each antibody). Anti-spectrin was used as an internal marker (Figure 2C–D). From E13.5, My32 detects three bands corresponding to adult type 2a, 2x and 2b which increase substantially in intensity from E15.5 showing that in mouse, My32 predominantly detects all three adult FMyHC isoforms. A band which co-runs with FMyHC 2a, but does not increase with embryonic stage, is detected by WB-MHCn. This is likely to be a neonatal MyHC but may be cross-reactivity with FMyHC 2a since this antibody also weakly detects the Adult MyHC in the marker track.

### Data analysis of Immunostaining and histological sections

Immunostaining patterns were established using a minimum of three separate staining runs per embryo and a mimimum of three separate embryos for each stage and strain. In many cases many more sections were stained. For the direct comparison of fast myosin and Igf-2 patterns, staining for both antibodies was carried out on carefully matched adjacent sections and repeated 3 or 4 times. Quantitation of fast myosin staining in wild type and mutant embryos and in neonatal skeletal muscles was achieved by assessing the intensity of staining (strong, weak or absent) in muscle fibres and by counting the number of myotubes in which staining was present in embryos and P1 pups). Only non-adjacent transversely sectioned muscle fibres were included in this analysis. At least 3000 fibres were counted for each data point and material from different embryos and different staining runs were included in the analysis to exclude experimental bias. Statistical analysis of these data was by Student t-test and ANOVA as indicated in the figure legends.

### Construction of MIG and MG transgenes

A bicistronic transgene (MIG, mCK-Igf-2- IRES2-EGFP) expressing insulin-like growth factor 2 (Igf-2) and green fluorescent protein (eGFP) from a muscle specific promoter (muscle creatine kinase, pmCK) and a control transgene (MG, mCK-IRES2-EGFP) expressing eGFP alone were constructed using a two stage process using a commercially obtained CMVpIRES-eGFP vector (pIRES2-EGFP, Clontech), p99a:5, a vector containing the coding region of Igf-2 (exons 4,5,6) (gift from Andrew Ward, University of Bath, UK [57]), and a full length muscle Creatine Kinase promoter containing vector (p3300MCKCAT) (a gift from Jean Buskin and Steve Hauschka, Seattle, USA [58]). The full length (p3300MCKCAT) promoter contains all three mCK regulatory elements and has been demonstrated to be equally expressed in all muscle fibre types [59]. To obtain MIG pIRES-EGFP (pIRES2-EGFP) was digested with AseI-XhoI to remove the CMV promoter (pIRES2-EGFP(-)) which, following modification of digested ends, was ligated sequentially with Igf2 (p99a:5) and the mCK promoter (p3300MCKCAT) via an intermediate construct pIGF2-IRES2-EGFP, to generate MIG. To generate the the control construct MG, the mCK promoter (p3300MCKCAT) was excised and ligated with a PstI-XmaI digestion of pIRES-EGFP (-). Plasmids with inserts were extensively characterised by restriction endonucleases mapping and DNA sequencing before transfection into skeletal muscle cells.

### Generation of MIG transgenic mice

MIG transgenic mice were generated by pronuclear injection. The transgene was removed from the vector by sequential digestion with EcoR1 and BspH1 followed by a Quiagen purification step. This fragment was microinjected into fertilized one-cell mouse embryos, derived from CBA × C57BL10 F_1 _matings. The embryos were implanted into CD1 pseudopregnant female mice. Previous attempts to generate MIG transgenic mice on C57BL10 and DBA × C57BL10 backgrounds resulted in very low numbers of MIG positive mice reaching term and all died within the first few days. These pups were fixed and characterised in comparison with their transgene negative littermates and with MG transgene positive pups to generate the data shown in Figure 4. All of these mice were morphologically normal and of the same size as their transgene negative siblings. To establish whether some of these animals were dying *in utero *we culled litters of MIG or MG injected embryos at E11.5, E13.5, E15.5 and E17.5 and compared litter size, resorption rate and transgene positivity between MIG and MG. Three litters were examined for each stage comprising a total of 117 (MG) and 120 (MIG) embryos. Litter size did not change with stage and was not significantly different between MIG and MG injected embryos (MIG 10 + 2.7; MG 9.75 + 1.4, not significantly different by Student t-test, p = 0.83). Resorption rate was also the same for both MG and MIG and did not alter with stage (MIG 3.25 + 0.45; MG 4 + 1.28, p = 0.36). The proportion of transgene positive animals generated was around 10% for both transgenes (MG 10.2%; MIG 9.3%). When allowed to reach full term, MIG mice are morphologically normal and do not exhibit respiratory difficulties at birth; however a majority die within the first few days probably through a failure to suckle. Recently we successfully fostered MIG + pups which survive, a more detailed characterisation of these mice will be published elsewhere. For genotyping, genomic DNA was isolated from placenta (embryos) or tail clips (P1 pups) and PCR carried out to amplify the fragment at the mCK and IGF2 junction (see Figure 2F). Transgene positive animals were then characterised using six different primers: mCKIgf-2 1 and 2 (across the mCK promoter and Igf-2 junction), Igf-2IRES 1 and 2 (across the Igf-2 and IRES junction) and GFP specific primers (MIGGFP 1 and 2).

### Primer sequence mCKIgf-2 1 (shown in Figure 3)

Forward: 5'- CTCCTCTATATAACCCAGGGGCAC-3'

Reverse: 5'- CCCCAACTGGGAAATCAAGAGAAG-3'

As a control MG transgenic mice were generated using the same procedure. For genotyping of these mice primers spanning the mCK-IRES junction (mCKIRES 1 and 2) and GFP-plasmid junction (MGGfp) were employed. These mice are viable and morphologically normal. The GFP expression pattern of both MG and MIG is consistent with previous reports for the full length muscle creatine kinase promoter (mCK) expression [57] and both transgenes are expressed in all muscle myotubes (see Figure 4A).

### Generation of C2MIG and C2MG myoblast cell lines

mCK-IGF2-IRES-gfp (MIG) and mCK-IRES-gfp (MG) constructs were transfected into C2C12 cell lines using Lipofectamine2000 (Invitrogen) followed by G418 selection. Single cell derived clones were then isolated and characterised for expression of the transgene (for methods refer to [60]. Two representative clones, C2MIG (H12) and C2MG (B9) were used throughout this study.

### Cell Culture and differentiation of C2MIG and C2MG cells

C2MIG and C2MG cells were plated out (1 × 10^4 ^cells/ml) into 8 well chamber slides (Nunc) and cultured for 24 hours in Dulbecco's Modified Essential Medium (DMEM, Gibco) supplemented with 10% Fetal Calf Serum (batch tested FCS; Sigma) before transferring either into differentiation medium (DMEM supplemented with 2% Horse Serum (batch tested HS; Sigma) or to fresh DMEM supplemented with 10% FCS. Cells were differentiated for 3 days. Differentiated cells were then fixed in 4% PFA and processed for immunohistochemistry (IHC) as described above. Each experiment presented was replicated out on 3 separate occasions and all experiments were carried out in duplicate. For each well 16 representative grid areas were counted per well (i.e. per replicate), representing a minimum of 2000 myoblasts per replicate. When cultured under these conditions, statistically, there was no difference in the total number of myotubes produced by C2MIG (H19) cells (15.3 ± 3.7 myotubes) per grid compared to C2MG (B9) (13.0 ± 2.8 myotubes per grid); no significant difference, Student t test, p = 0.47.

Plating efficiency analysis was carried out by plating cells (C2MG or C2MIG) at a density of 10^4 ^cells/90 cm dish followed by incubation for 6 hours and fixation and staining in Leishman's methanol stain (see [31], for method). Cloning analysis was carried out by plating cells at 10^3^/plate and incubating for 5 days before fixation and staining in Leishmans'stain as before. Plating efficiency and colony formation assays carried out on C2MG (B9) and C2MIG (H19) cells showed no significant difference between the two lines (Student t test: p = 0.23 and p = 0.60 respectively).

### ShRNA Interference

#### Constructs

ShRNA constructs were generated against Gfp (RNAi-*Gfp*) and Igf-2 (RNAi-*Igf-2*) respectively using the pSHAG RNAi vector system described by [61]. Unique 29bp hairpin sequences were inserted into a HIND III site in the pSHAG vector. A Not1 site is located upstream of the U6 promoter region. ShRNAi inserts could therefore be detected by the presence of a larger plasmid band following Hind111 digestion of the construct. The presence of the ShRNA insert was confirmed by a double restriction enzyme digest using HIND111 and Not1. Sequences for the Gfp and Igf-2 RNAi inserts were as follows:

**Forward: RNAi eGFP: **5'-AAC TTC AGG GTC TAG GTG GGA AGC TTG CTA CTT ATG GCA AGC TGA CTC TGA AGT TCA TTT TTT T-3'

**Reverse: RNAi eGFP: **5'-GAT CAA AAA AAT GAA CTT CAG AGT CAG CTT GCC ATA AGT AGC AAG CTT CCC ACC

**Forward: RNAi Igf-2: **5'-CGA CGG TTG GCA CGG CTT GGA GGC CTT CGA GTC GTG CTA ACC GTC GCA GTT TTT T-3'

**Reverse: RNAi Igf-2:**5'-GAT CAA AAA ACT GCG ACG GTT AGC ACG ACT CGA AGG CCT ACC AAG CCT TCA AGC CGT GCC AAC CGT CGC G-3'

### ShRNA Transfection

C2MIG and C2MG skeletal muscle cells were transfected using an optimized modification of the manufacturers instructions for use with lipofectamine 2000 (Invitrogen). Cells were incubated in DNA/lipofectamine 2000 for 24 hours before being plated out at 1x10^4 ^cells/ml into DMEM+10%FCS for 24 hours. Cells were switched into differentiation medium (DMEM supplemented with 2% batch tested Horse Serum (Sigma)) for 3 days before being either fixed in 4% PFA followed by immunohistochemistry, or subject to RNA extraction for RT-PCR analysis or protein extraction for western blotting.

### RT-PCR

Verification of Igf-2 RNAi knockdown was achieved by semi-quantitative RT-PCR ([31] method modified for non-radioactive detection). Total RNA was extracted from cell cultures using TRIZOL and following the manufacturer's instructions (Gibco; Invitrogen Life Technologies). First strand cDNA was generated using a SuperScript II first-strand synthesis system for RT-PCR (Invitrogen Life Technologies). The linear range of product amplification was established for each PCR primer set used (Igf-2, GFP and α-tubulin). An equalisation step was carried out in which all cDNA's were diluted in series and subject to PCR for α-tubulin. GFP and Igf-2 were amplified using these equalised concentrations. For all primer sets amplification was preceded by one 3 minute cycle at 94°C and concluded with a final extension step at 72°C for 5 minutes. Annealing temperatures and number of cycles is indicated below. To ensure detection of *Gfp *RNA message only, RNA was prepared as above except that, prior to being reverse transcribed, it was DNase treated (Invitrogen Life Technologies) following manufactures guidelines. Results were visualised by electrophoresis (1% agarose gel), stained with ethidium bromide and quantified by densitometry.

*Igf2 Primers (31 cycles; annealing temperature 56°C)*

Forward: 5' AGT CGA TGT TGG TGC TTC TC -3'

Reverse: 5' TGA TGG TTG CTG GAC ATC TC - 3'

*α-tubulin Primers (19cycles; annealing temperature 55°C)*

Forward: 5' AGA TGC CAA GTG ACA AGA CC - 3'

Reverse: 5' AGA TGG CCT CAT TGT CTA CC - 3'

*Gfp Primers (22 cycles; annealing temperature 55°C)*

Forward: 5' AGG ACG ACG GCA ACT ACA AG - 3'

Reverse: 5' CTG GGT GCT CAG GTA GTG GT - 3'

### Protein Preparations and Western Blot Analysis

Whole cell extracts were taken from cells differentiated for 3 days using RIPA buffer and protease inhibitors (Roche) mix and stored at -80°C. Protein (15 μg) was electrophoresed on 10% SDS-PAGE and then transferred to nitrocellulose membranes (Amersham). ECL detection (Pierce Endogen Hyclone) was carried out in accordance to manufactures recommendations. Antibodies used were as follows: Igf-2 and fast myosin clone My32 (as described above); α-tubulin IgG_1 _(1/1000, Sigma); spectrin (1/00, Novocastra); neonatal myosin (Wb-MHCn 1/250, Novocastra) and Goat anti-mouse IgG-HRP (1/2000, Santa Cruz Biotechnology).

## Authors' contributions

DM participated in the Igf-2 and FMyHC immunostaining patterns and carried out the ShRNAi and MIG *in vitro *experiments. She also participated in the immunostaining and analysis of Mat Di and MIG/MG embryos and pups. TT generated MIG and MG constructs, C2MG and C2MIG cell lines and transgenic mice. She also participated in the characterisation of these mice and in analysis and immunostaining of MIG/MG pups. Both DM and TT contributed to the writing of the manuscript. LKJS participated in the immunostaining and scoring of MatDi, MIG and MG embryos. DM and LKJS participated in photography of the work. DM, TT and LKJS all contributed to the design of the study. JS conceived of the study participated in its design and coordination. She also wrote and revised the manuscript. JS also carried out the Igf-2 peptide blocking, participated in the immunostaining experiments and characterisation of C2MG and C2MIG cell lines and contributed to photography, scoring and data analysis. All authors read and approved the final manuscript.
